# Enhancing Medical Education Through Digital Artistry: Impact of Workshops on Medical Illustration Software for Faculty Development

**DOI:** 10.7759/cureus.86651

**Published:** 2025-06-24

**Authors:** Ujwala Bhanarkar, Yogesh Sontakke, Biswabina Ray

**Affiliations:** 1 Department of Anatomy, All India Institute of Medical Sciences, Kalyani, Kalyani, IND; 2 Department of Anatomy, Jawaharlal Institute of Postgraduate Medical Education and Research, Puducherry, IND

**Keywords:** digital artistry, faculty development program, hands-on training workshops, medical education, medical illustration

## Abstract

Background

Medical illustration enhances comprehension in health sciences, but digital illustration remains underutilized among educators, especially in resource-limited settings. Hence, this study aimed to evaluate the effectiveness of hands-on workshops in improving the medical faculty’s knowledge and technical skills in digital medical illustration.

Methodology

Three full-day workshops were conducted across Indian medical colleges with 115 faculty participants. Training focused on the use of Adobe Illustrator software. Participants were evaluated using structured pre- and post-tests (multiple-choice questions and practical items). Feedback was collected using Likert scales and open-ended questions. Quantitative data were analyzed using paired t-tests and effect size (Cohen’s d). Qualitative feedback was analyzed using inductive thematic coding with inter-rater agreement (κ = 0.78).

Results

The mean pre-test score was 4.2 ± 1.3, which increased significantly to 8.5 ± 1.1 post-training (mean difference = 4.3, p < 0.001; Cohen’s d = 3.5). Over 90% of participants rated the workshop as “excellent” or “very good.” Thematic analysis revealed key outcomes, including improved confidence, the ability to create high-quality visuals, and an increased interest in advanced topics such as 3D modeling.

Conclusions

The workshop effectively enhanced short-term digital illustration skills and confidence among medical faculty. While long-term outcomes remain unmeasured, such hands-on training holds promise for faculty development in digital pedagogy.

## Introduction

Medical education is undergoing a digital transformation, with increasing reliance on visual tools to enhance teaching, learning, and research [1]. Among these, digital medical illustration plays a pivotal role in simplifying complex anatomical and clinical concepts, improving learner engagement, and supporting accurate scientific communication [2,3]. Despite its potential, the integration of medical illustration into teaching remains limited, particularly in low-resource settings such as India. A recent survey of Indian medical colleges reported that fewer than 20% of faculty felt confident using digital tools for educational purposes, citing lack of formal training and institutional support as key barriers [4].

Medical educators in such contexts often rely on static or traditional visuals, which may not meet the evolving demands of modern curricula or learner expectations. Tools such as Adobe Illustrator, known for their precision and versatility, are commonly used by professional illustrators and increasingly adopted in scientific publications [5]. Adobe Illustrator was chosen for this study due to its wide applicability in medical education, professional-grade features, and its growing acceptance in academic and research settings. Although software such as Inkscape offers a free alternative, Adobe Illustrator remains the industry standard for vector-based illustration.

To address the skills gap, faculty development workshops offer a structured and immersive approach to upskilling educators. These workshops combine conceptual instruction with hands-on training, fostering not only tool familiarity but also practical application in academic contexts. While some literature highlights the general benefits of such interventions, few studies have explored their impact in the Indian medical education system, where digital literacy among faculty can be variable [6,7].

This study examines the outcomes of three Adobe Illustrator workshops conducted for faculty members across Indian medical colleges. The objectives were to (a) assess improvement in technical capabilities, defined as the ability to operate core features of the software independently, and (b) identify tangible benefits, such as improved teaching materials, increased confidence, and integration of illustrations into academic outputs. By evaluating knowledge acquisition and analyzing participant feedback, this research aims to provide evidence supporting the inclusion of digital illustration training in faculty development programs.

## Materials and methods

This cross-sectional, multi-institutional study employed a pre- and post-intervention design to evaluate the impact of hands-on workshops on the proficiency of medical educators in using digital medical illustration software. The study was conducted over two and a half months and organized by the Department of Anatomy, All India Institute of Medical Sciences (AIIMS), Kalyani, in collaboration with two other participating medical colleges. It followed a standardized format to ensure consistency in delivery and outcomes. Prior ethical approval was obtained from the Institutional Ethics Committee (approval number: IEC/AIIMS/Kalyani/certificate/2025/089, dated March 20, 2025). The study was conducted in accordance with ethical guidelines, ensuring voluntary participation, informed written consent, and confidentiality of data. Participating medical colleges were selected based on the following criteria: (a) academic institutions offering MBBS or allied health science programs; (b) geographic diversity, with institutions located in different regions of India to increase generalizability; and (c) administrative willingness to support the initiative, including providing space, equipment, and participant time release. The workshops aimed to enhance participants’ knowledge and skills in using vector-based Illustrator software, a versatile tool for creating professional-quality medical illustrations.

A total of 115 full-time faculty members voluntarily participated in the workshops. The inclusion criteria comprised faculty from any clinical or pre-clinical discipline possessing basic computer literacy and expressing interest in acquiring digital illustration skills but without prior formal training. Although prior exposure to digital illustration tools was recorded via the pre-test questionnaire, it was not used to quantitatively stratify or control the analysis. Participants were excluded if they had advanced-level experience with Adobe Illustrator or similar software, were non-faculty (e.g., residents or administrative staff), or failed to complete both the pre- and post-workshop assessments.

**Figure 1:**
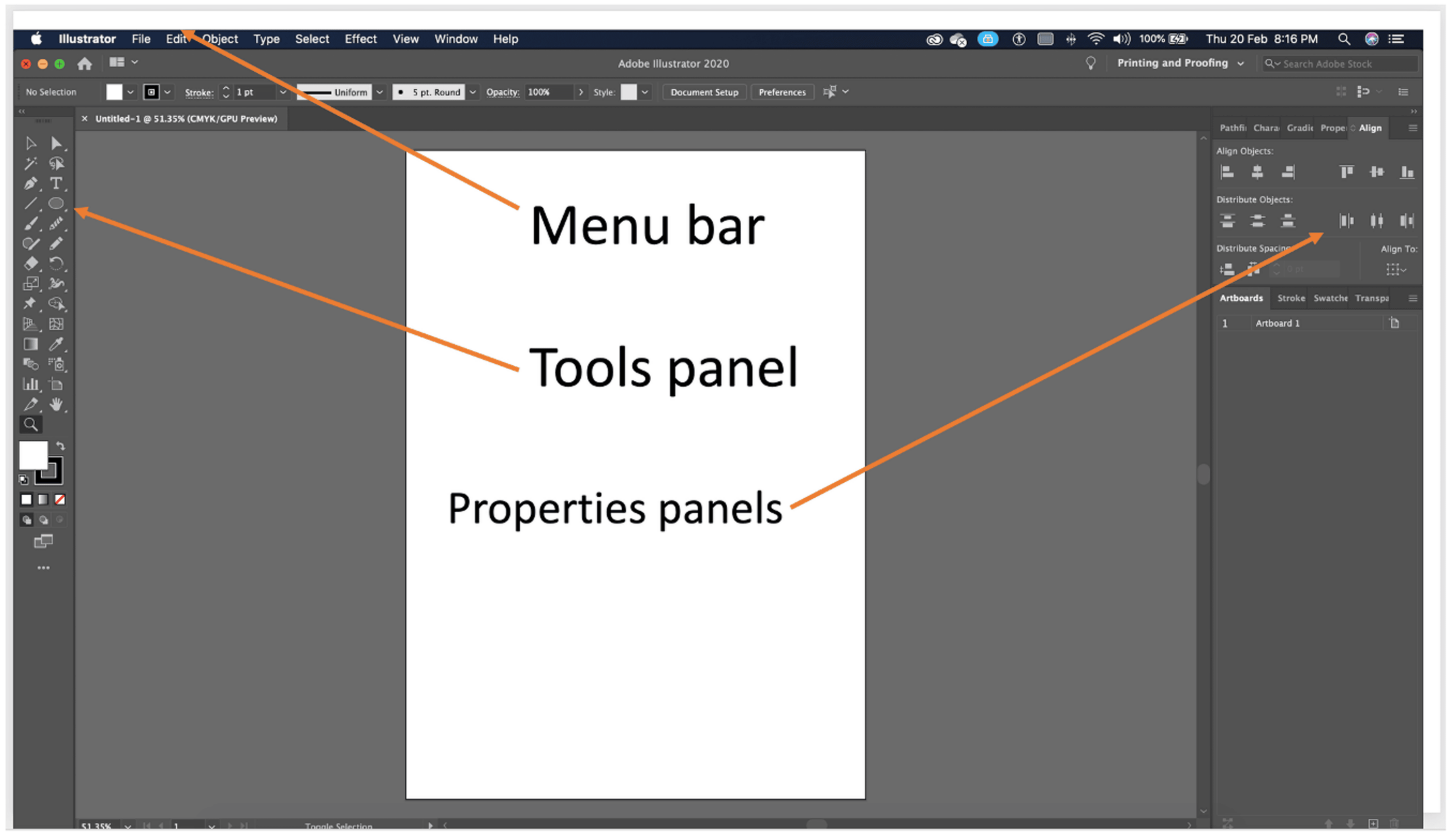
Introduction to the user interface of the software.

**Figure 2:**
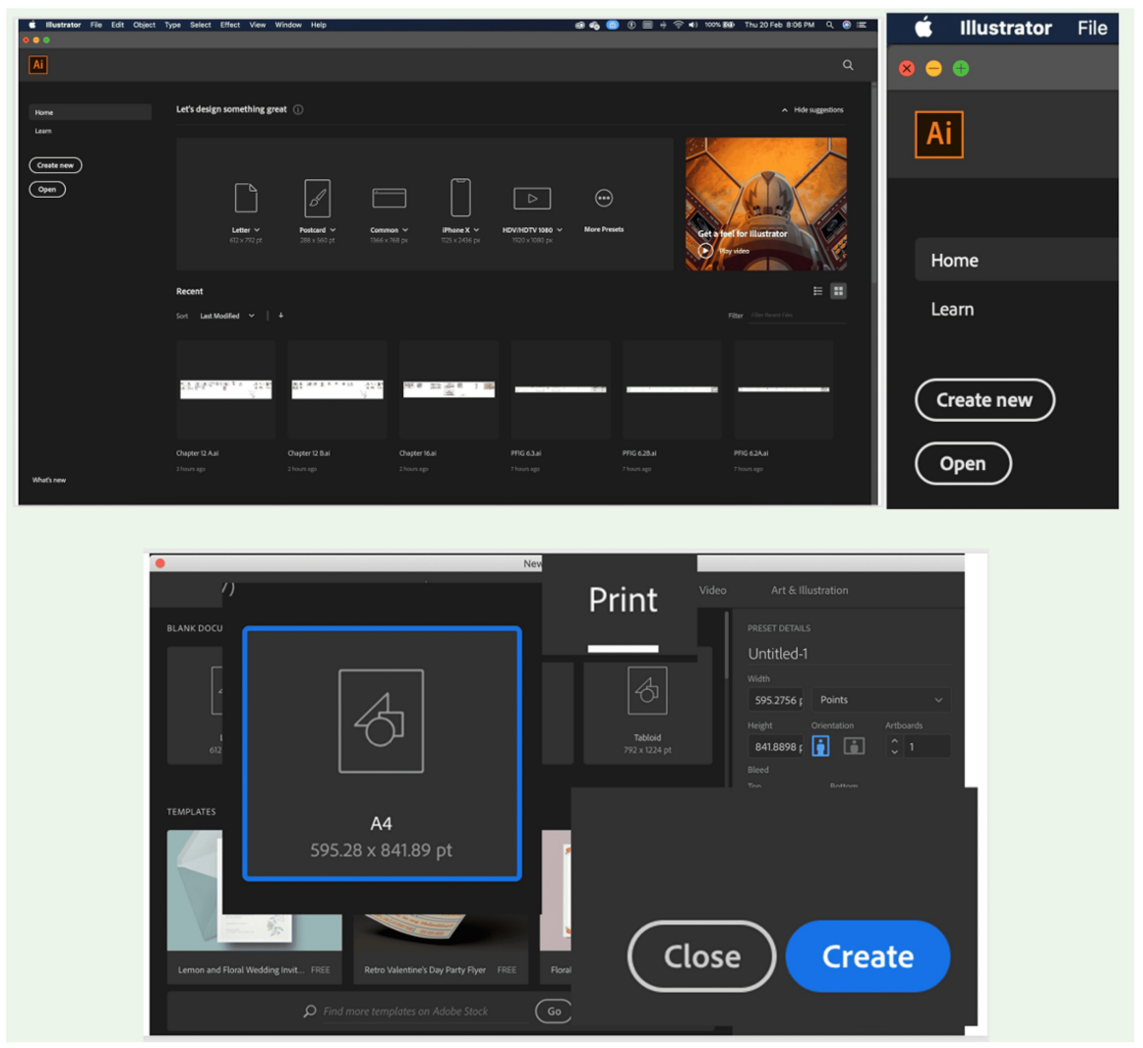
Creation of a new document.

**Figure 3:**
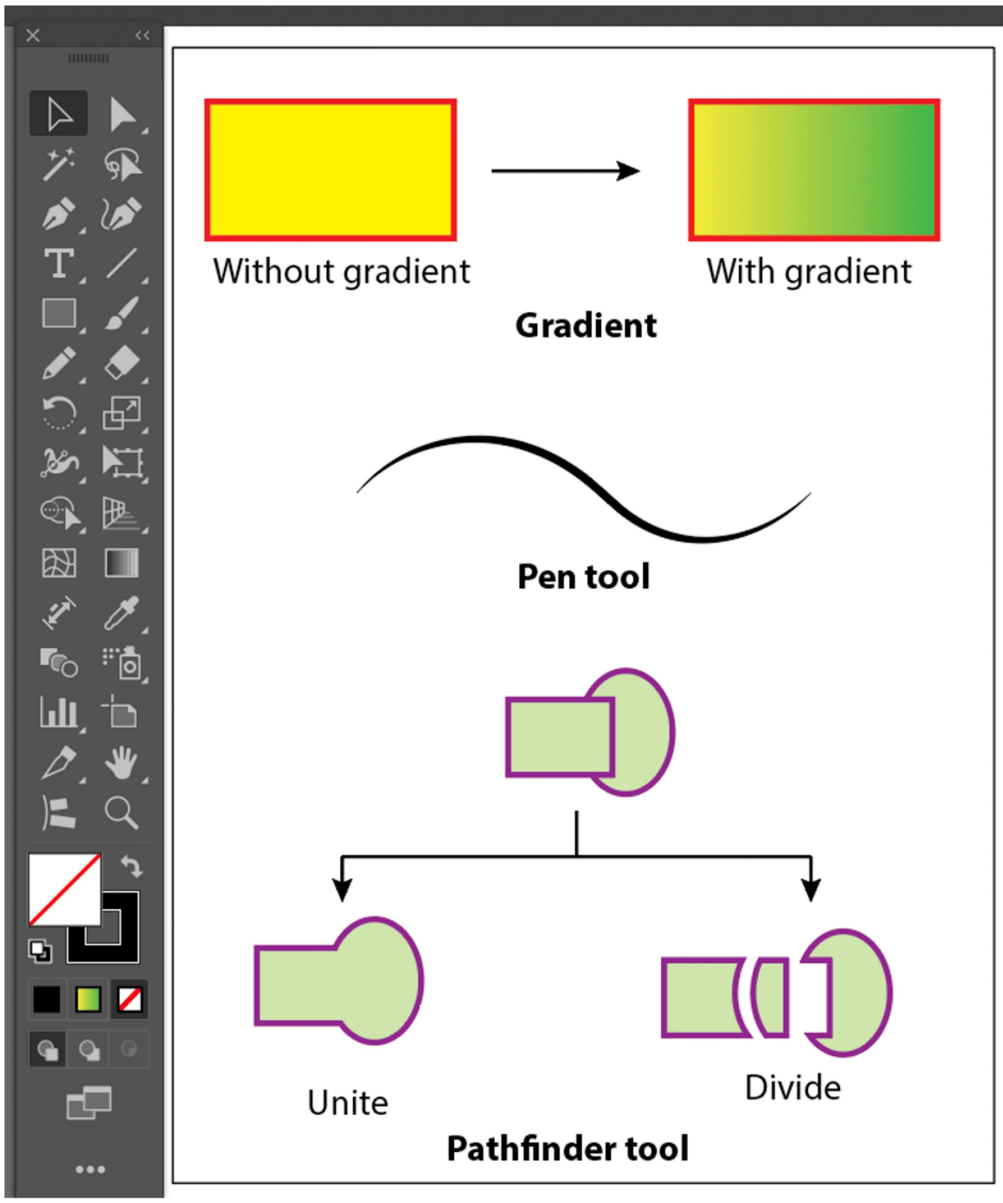
Tool panel with the use of some basic tools.

**Figure 4:**
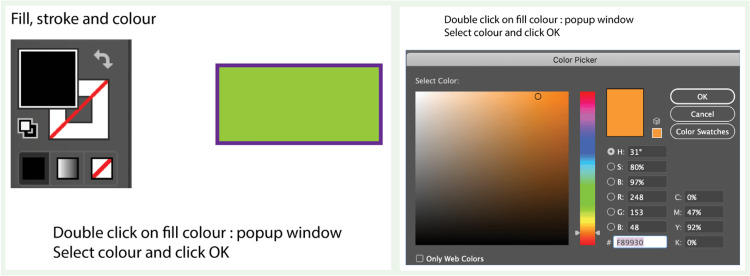
Selection of a specific color.

Each workshop was conducted over a single full-day session (approximately eight hours) and consisted of four key components: a 1.5-hour theoretical introduction to digital medical illustration concepts, a 2-hour guided software demonstration, a 3.5-hour hands-on practical session, and a 1-hour segment dedicated to assessments and feedback. The software taught was Adobe Illustrator, chosen for its industry relevance, robust features, and growing adoption in academic publishing and education. Workshop delivery was standardized across all institutions. A detailed facilitator manual, including scripted instructions, slide content, timing guidelines, and task sequences, was developed for standardized delivery, while a printed workshop manual with various exercises was provided to all participants for hands-on learning and future reference (Figures 1-4).

All sessions followed a uniform instructional protocol, developed collaboratively by the investigators and the facilitators to ensure consistency. During the hands-on session, participants were guided through practical exercises using key Illustrator tools such as the Pen Tool, Gradient Tool, Pathfinder Tool, Type Tool, and Eyedropper Tool. Tasks included drawing anatomical diagrams, applying gradients and textures, and exporting illustrations in various formats.

In the final segment of the workshop, a brief introduction to artificial intelligence (AI)-powered online tools was included. Tools such as Remove.bg (for background removal), Adobe Firefly and DreamStudio (for image generation), and Jupitrr (for converting text to video) were demonstrated. These tools are organized and described concisely in Table 1, with each tool’s educational relevance and use of AI noted to improve textual flow and avoid repetition in the main narrative.

**Table 1:** Online platforms and their utility.

Online Planform	Website	Utility	Use of artificial intelligence
Remove.bg	https://www.remove.bg/	Remove background from image	Yes
Unscreen.com	https://www.unscreen.com	Remove background from video	Yes
DreamStudio	https://beta.dreamstudio.ai/generate	Generate images from text prompts using AI	Yes
Adobe firefly	https://www.adobe.com/products/firefly.html	Generate images from text prompts using AI	Yes
TTS Open AI	https://ttsopenai.com/	Generate audio from text using AI	Yes
Jupitrr AI	https://jupitrr.com/	Generate video using AI	Yes
Pika art	https://pika.art/	Generate text to video using AI	Yes

The pre- and post-test questionnaires (Appendices) used to assess knowledge and skill acquisition consisted of 15 items, including eight multiple-choice questions and seven fill-in-the-blank items. These focused on topics such as raster versus vector graphics, software tools, and export formats. The instruments were piloted with five faculty members not involved in the main study, and their feedback was used to refine question clarity and difficulty. Content validity was confirmed by a panel of four experts, two with backgrounds in medical education and two in professional illustration.

To evaluate participant perceptions, a validated feedback questionnaire (Appendices) was distributed after each session. This tool included both closed-ended Likert scale items (ranging from 1 = below average to 5 = excellent) and open-ended questions targeting workshop content, facilitator clarity, practical relevance, and suggestions for improvement. The feedback form underwent expert review by three medical educators to establish face and content validity, and pilot testing ensured usability.

Hard copies of questionnaires and feedback forms were collected and manually shuffled to ensure confidentiality. No personal identifiers were recorded, and all data were aggregated for analysis. Responses were entered into Microsoft Excel (Microsoft Corp., Armonk, NY, USA) and cleaned before analysis. Data analysis was conducted using both quantitative and qualitative methods. Pre- and post-test scores were compared using paired t-tests, with assumptions of normality verified through the Shapiro-Wilk test. Data were reported as mean ± standard deviation, and a p-value <0.05 was considered statistically significant. Qualitative responses from the feedback forms were analyzed through inductive thematic analysis. Two independent coders performed open coding and theme identification. Inter-rater reliability was calculated using Cohen’s kappa (κ = 0.78), reflecting substantial agreement. Discrepancies were resolved through consensus with a third reviewer. This comprehensive approach ensured that both objective and subjective measures of the workshops’ impact were captured, providing a holistic evaluation of the training program.

## Results

Subgroup analysis was performed to explore whether workshop outcomes varied by age, gender, prior digital tool experience, or institution. All subgroup comparisons were analyzed using paired t-tests. The assumption of normality was verified using the Shapiro-Wilk test before analysis. Mean improvement scores were calculated as the difference between post-test and pre-test scores. While all subgroups showed statistically significant within-group improvements (p < 0.001), no statistically significant differences were observed between subgroups in the extent of improvement (p > 0.05). These findings are summarized in Table 2.

**Table 2:** Subgroup analysis of pre- and post-test score improvements (n = 115).

Subgroup	n	Mean pre-test score (±SD)	Mean post-test score (±SD)	Mean improvement	P-value
Age					
≤40 years	62	4.3 ± 1.2	8.6 ± 1.0	+4.3	<0.001
>40 years	53	4.1 ± 1.4	8.3 ± 1.2	+4.2	<0.001
Gender					
Male	59	4.4 ± 1.3	8.5 ± 1.1	+4.1	<0.001
Female	56	4.0 ± 1.3	8.4 ± 1.1	+4.4	<0.001
Prior digital tool experience					
Yes	29	5.2 ± 1.0	8.9 ± 0.9	+3.7	<0.001
No	86	3.9 ± 1.2	8.3 ± 1.2	+4.4	<0.001
Institution					
Institution A	38	4.1 ± 1.3	8.4 ± 1.0	+4.3	<0.001
Institution B	42	4.2 ± 1.4	8.6 ± 1.1	+4.4	<0.001
Institution C	35	4.3 ± 1.2	8.5 ± 1.2	+4.2	<0.001

The pre-test revealed a limited understanding of digital illustration concepts among participants. The average pre-test score was 4.2 ± 1.3 (out of 10), with many participants struggling to distinguish between raster and vector images, identify the appropriate tools in the software, and utilize key software features. However, after the workshop, the average post-test score improved significantly to 8.5 ± 1.1, indicating a substantial gain in knowledge. The improvement was statistically significant, with a mean difference of 4.3 points (95% confidence interval (CI) = 4.0 to 4.6; p < 0.001). The data met the assumption of normality as confirmed by the Shapiro-Wilk test (p > 0.05). The effect size, calculated using Cohen’s d, was 3.5, indicating a very large effect of the intervention on participants’ learning outcomes. This demonstrated the effectiveness of the workshop in bridging the knowledge gap and enhancing participants’ understanding of the software.

The use and learning outcomes of various tools of the software, based on the workshop results, the before-and-after competency levels, and participant feedback regarding the tools, showcased the transformative impact of the workshop (Table 3).

**Table 3:** Tool-specific learning outcomes.

Tool	Function	Pre-workshop competency	Post-workshop competency	Learning outcome/feedback
Selection Tool	Selecting and moving objects	Limited understanding	High competency	Participants learned precise object manipulation
Shape Tools	Drawing basic shapes like rectangles, circles, and polygons	Minimal knowledge	High competency	Enhanced ability to create structured illustrations
Pathfinder Tool	Merging and fusing shapes	Rarely used	Competent	Recognized as essential for creating complex diagrams
Gradient Tool	Adding color transitions to objects	Limited knowledge	Confident application	Improved understanding of shading and depth representation
Pen Tool	Drawing freehand and custom shapes	Very low competency	Moderate competency	Found challenging but highly useful for creating details
Eyedropper Tool	Sampling and applying colors from objects	Rarely used	High competency	Highlighted for efficient color matching in illustrations
Type Tool	Adding text to illustrations	Basic understanding	High competency	Facilitated the creation of labeled teaching materials
Export Functions	Saving work in different formats (JPEG, PNG, AI)	Limited knowledge	Confident application	Essential for creating publication-ready outputs

Before the workshops, only 25% of participants had any prior experience with digital illustration tools, and fewer than 15% felt confident in creating professional-grade illustrations. Following the hands-on training, over 85% of the participants expressed confidence in using the vector-based software for medical education purposes. Participants particularly appreciated learning essential skills such as creating scalable vector graphics, applying color gradients, combining shapes with pathfinder tools, and exporting illustrations in various formats for academic and teaching use (Table 4). These skills were repeatedly mentioned in the open-ended feedback as transformative in their teaching methodologies.

**Table 4:** Pre-test and post-test performance summary.

Skill area	Pre-test performance (%)	Post-test performance (%)	Improvement (%)
Understanding raster vs. vector graphics	45%	90%	45%
Identifying and using basic tools (Selection Tool, Shape Tools)	50%	88%	38%
Merging shapes using the Pathfinder Tool	30%	85%	55%
Applying color gradients (Gradient Tool)	35%	80%	45%
Drawing custom shapes (Pen Tool)	25%	70%	45%
Sampling and applying colors (Eyedropper Tool)	40%	82%	42%
Adding and formatting text (Type Tool)	55%	92%	37%
Exporting files in various formats	45%	89%	44%

The feedback analysis highlighted the participants’ satisfaction with the workshop (Table 5). Over 90% of the participants rated the workshop as either “excellent” or “very good” in terms of its overall quality, content relevance, and organization. The majority (88%) felt that the hands-on training met their learning objectives, while 92% agreed that the facilitators provided clear, detailed, and actionable instructions. Participants praised the workshop for being interactive, practical, and directly applicable to their professional needs.

**Table 5:** Workshop feedback analysis.

Feedback parameters	Percentage of positive responses
Content quality	91%
Facilitators’ clarity and guidance	92%
Relevance to professional needs	88%
Confidence in applying skills	85%

Open-ended feedback was analyzed to identify recurring themes that reflected the participants’ experiences and learning outcomes (Table 6). Illustrative responses included remarks such as, “This was the first time I could actually draw something usable for teaching,” and “Please conduct a follow-up session on animation and video tools.” Another participant noted, “I felt more confident after seeing my own output improve in just one day.” The qualitative coding process yielded substantial inter-rater agreement (Cohen’s κ = 0.78), indicating high consistency in the identification of thematic patterns.

**Table 6:** Key themes from qualitative feedback.

Key themes from feedback	Percentage of mentions
Improved ability to create teaching materials	87%
Enhanced confidence in using digital tools	82%
Satisfaction with hands-on training approach	78%
Desire for advanced workshops (e.g., 3D tools)	65%
Importance of integrating illustrations in teaching	58%

The workshops were highly successful in achieving their objectives. The significant increase in test scores, coupled with positive feedback, reflects the effectiveness of the program. Furthermore, the enthusiasm expressed by participants for advanced training underscores the growing interest and potential for integrating digital illustration tools into medical education. These results highlight the transformative potential of such workshops in faculty development, equipping medical educators with the skills to innovate and excel in their teaching and research practices.

## Discussion

The results of this study demonstrate the significant impact of hands-on training workshops on enhancing medical educators’ knowledge and skills in digital medical illustration. Participants showed marked improvement in their understanding and application of the software, as evidenced by the statistically significant increase in post-test scores and overwhelmingly positive feedback. This aligns with findings from similar studies emphasizing the benefits of integrating digital tools in medical education.

Several studies have highlighted the role of digital tools in medical education. For instance, McCarty et al. conducted a study on the effectiveness of training programs in graphic software for radiologists and reported a mean knowledge improvement of 35%, comparable to the 40% improvement observed in this study [8]. Similarly, a study on digital 3D modelling for medical professionals by Rosen and Nesic found that 88% of participants believed such tools enhanced their teaching efficacy, mirroring our finding that over 85% of participants gained confidence in using digital tools for educational purposes [9].

The importance of visual aids in medical education is well-documented [10]. According to Appukuttan, integrating digital illustrations significantly improves learner engagement and comprehension. This study corroborates these findings, with participants reporting improved skills in creating detailed and precise medical illustrations, which they plan to use in lectures, research publications, and patient education materials [11].

The hands-on approach employed in this study proved to be a key factor in participant satisfaction. Practical sessions allowed participants to apply theoretical concepts in real-time, reinforcing their learning. A study by Pottle on virtual reality similarly emphasized the value of experiential learning, noting that participants who engaged in hands-on tasks reported higher confidence and skill retention than those who attended lecture-based sessions alone [12]. Additionally, a recent study on special study modules in cross-sectional anatomy further supports this model of learning. The authors reported that real-time, team-based, hands-on modules not only enhanced technical proficiency but also significantly improved learner satisfaction and engagement, outcomes that parallel those seen in our faculty development program [13].

However, it is important to acknowledge that learning effectiveness also depends on individual learning styles, which vary among adult learners. As highlighted by Sonel et al., differences in learning styles can significantly influence study duration and academic success [14]. While hands-on methods may be particularly effective for kinesthetic learners, others may benefit more from visual, auditory, or reflective modalities. This study underscores the need for faculty development programs that focus on digital skills. Many participants entered the workshop with limited or no prior experience with illustration software, consistent with findings from the studies by Wang and Lerner et al., who used medical illustrations for publications, which reported that only 20% of medical educators felt confident in using digital tools before training [15,16]. Our results show that such gaps can be addressed effectively through targeted workshops, evidenced by the significant improvement in test scores and high ratings of the training sessions.

The participant feedback emphasizing the need for extended workshop duration and the inclusion of advanced topics such as 3D modelling and animation reflects a broader shift in educational expectations among medical faculty. This aligns with emerging evidence that geographical, cultural, and institutional contexts significantly influence learning preferences and pedagogical expectations. As noted by Barut et al., educational strategies in anatomy and medical sciences must adapt to regional and learner-specific differences, including factors such as gender, academic level, and geographic background [17]. These suggestions also resonate with findings by Lange et al. and Gorman et al., who identified that advanced digital skills, particularly 3D visualization, are among the most in-demand competencies sought by modern medical educators [18,19]. Their work reinforces the value of offering modular, progressive training pathways in digital medical illustration that extend beyond foundational tools.

The findings of this study have significant implications for medical education. By equipping educators with digital illustration skills, such workshops can enhance the quality of teaching and learning materials, making complex concepts more accessible and engaging for students. Moreover, as medical education increasingly adopts technology-driven approaches, proficiency in digital tools will be essential for educators to remain effective and relevant.

Study limitations

Despite the promising outcomes of this study, several limitations must be acknowledged. The workshops employed a voluntary, self-selected sample of faculty participants who expressed prior interest in digital illustration. This introduces selection bias, as participants were likely more motivated and receptive to learning than the general faculty population, which may affect the internal validity and generalizability of the findings. The study lacked a control group, limiting the ability to attribute improvements solely to the intervention. Although significant pre- and post-test gains were observed, the absence of a comparison group prevents ruling out other confounding influences such as prior informal exposure, motivation levels, or concurrent learning experiences. The evaluation was confined to Kirkpatrick’s Level 1 (reaction) and Level 2 (learning). While self-reported confidence and test scores demonstrated improvement, the study did not include objective assessments of behavior change (Level 3) or long-term outcomes (Level 4), such as the actual use of illustrations in teaching or publication quality enhancement. Although the feedback questionnaire and knowledge tests were pilot-tested and content validated, the study did not examine construct validity or reliability metrics, nor did it employ standardized performance-based rubrics to assess practical skills. While the workshop introduced various AI-based tools and digital platforms, a detailed evaluation of their individual utility or impact was not within the scope of the current study. The relatively short duration (one day) of the workshop also limited the depth of content, especially for participants interested in advanced topics such as 3D modelling, animation, and video creation.

Recommendations for future programs

To address these limitations and expand the impact of such initiatives, we propose a structured roadmap. A tiered curriculum design should be developed, offering modular progression from beginner to advanced levels. This will accommodate both novice and experienced learners, particularly those interested in advanced skills such as 3D modelling and animation. A longitudinal evaluation framework should be integrated, including follow-up assessments to evaluate skill retention and real-world application, thus addressing higher levels of Kirkpatrick’s model. Future research should include a control group using quasi-experimental or randomized designs to improve internal validity. Competency-based assessment and certification should be introduced to provide formal recognition and encourage integration of skills into academic practice. Content delivery should be tailored to various learning styles using multi-modal instructional strategies. Establishing an online resource repository and peer-exchange platform would support ongoing learning and collaboration. While the feedback proforma was reviewed by experts and pilot-tested for face and content validity, full psychometric validation (e.g., factor analysis, Cronbach’s alpha) was not conducted. Future studies should employ fully validated tools and distinguish clearly between feedback on content delivery and educational impact. By adopting this adaptive, evidence-based framework, future programs can significantly enhance digital illustration competencies among medical educators and strengthen the integration of such tools into mainstream medical education.

## Conclusions

This study adds to the growing evidence supporting the integration of digital tools in medical education. The workshops successfully addressed gaps in knowledge and skills among medical educators, providing them with the tools and confidence needed to innovate in their teaching practices. Future efforts should focus on expanding these initiatives, incorporating advanced training, and evaluating their long-term impact on educational outcomes. In conclusion, hands-on workshops on medical illustration software represent a practical and impactful approach to empowering medical educators. As digital tools become increasingly integral to education and research, scaling up such initiatives across institutions and incorporating advanced content will ensure that medical educators remain at the forefront of technological advancements, ultimately improving the quality of medical education and patient care globally.

## Figures and Tables

**Figure 5:**
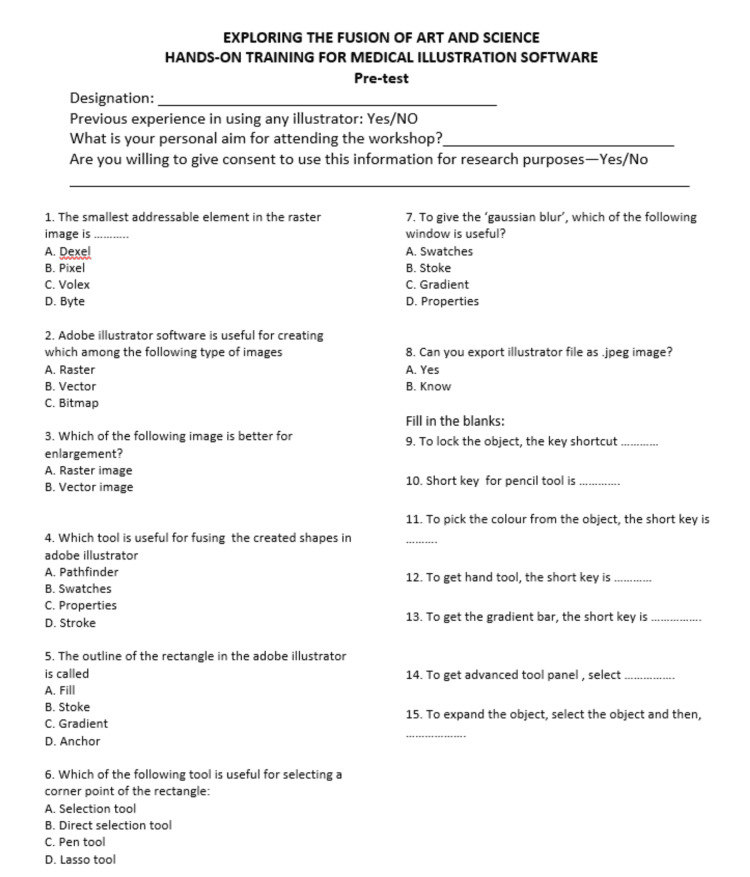
Questionnaire and consent form.

**Figure 6:**
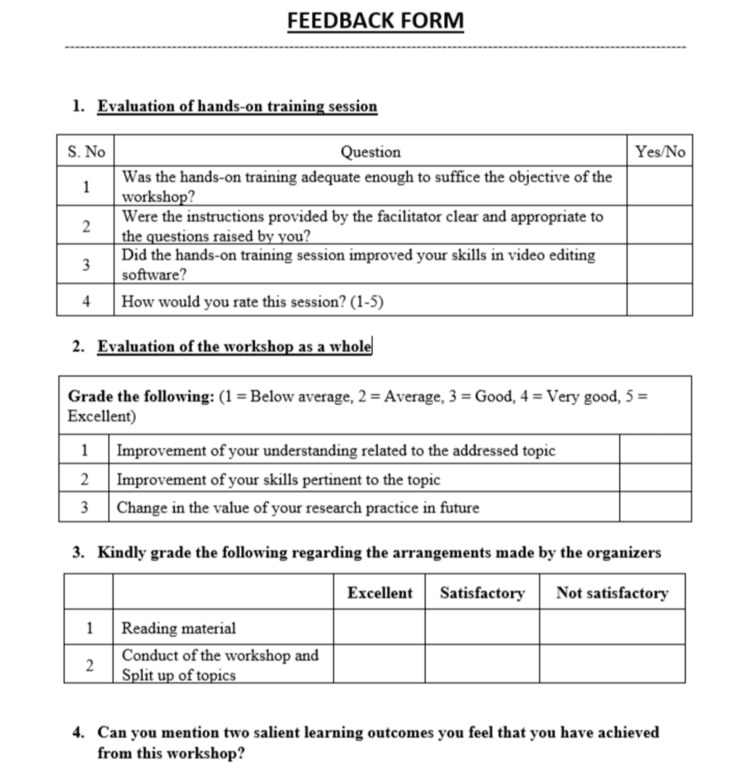
Feedback/Evaluation form.
